# Timing the Origin of *Cryptococcus gattii* sensu stricto, Southeastern United States

**DOI:** 10.3201/eid2411.180975

**Published:** 2018-11

**Authors:** Shawn R. Lockhart, Chandler C. Roe, David M. Engelthaler

**Affiliations:** Centers for Disease Control and Prevention, Atlanta, Georgia, USA (S.R. Lockhart);; Northern Arizona University, Flagstaff, Arizona, USA (C.C. Roe);; Translational Genomics Research Institute, Flagstaff (D.M. Engelthaler)

**Keywords:** Cryptococcus gattii, Bayesian analysis, fungi, yeast, emergence, southeastern United States, United States

## Abstract

We conducted molecular clock analysis of whole-genome sequences from a set of autochthonous isolates of *Cryptococcus gattii* sensu stricto from the southeastern United States. Our analysis indicates that *C. gattii* arrived in the southeastern United States approximately 9,000–19,000 years ago, long before its arrival in the Pacific Northwest.

The *Cryptococcus gattii* species complex consists of >4 major subtypes (VGI–VGIV), which are now considered to be different species (*1*). *C. gattii* species complex is often described as native to tropical and subtropical regions, but recent reports have shown that it is more cosmopolitan than previously thought (*2*). In North America, the emergence of *C. deuterogattii* (VGII) in the Pacific Northwest of Canada and the United States generated a great deal of interest in the study of *C. gattii* species complex in this area (*3*). Although this emergence was the impetus for the study of *C. gattii* species complex in the United States, this pathogen has actually been known and documented in the United States for multiple decades, especially in southern California (*4*). Despite its occurrence in the Pacific Southwest, the emergence in the Pacific Northwest is thought to be quite recent. Recent work using Bayesian evolutionary analysis by sampling trees generated using BEAST software showed that the emergence of *C. deuterogattii* in the Pacific Northwest was a recent event, occurring within the last 60–100 years (*5*).

Historical reports have described the presence of *C. gattii* species complex in the southeastern United States, where documented clinical cases are rare. However, these cases are often unacknowledged by literature reviews because they occurred when *C. gattii* could only be detected as a unique serotype of *C. neoformans*, before it became known as a separate species (*6*). Although published reports have been rare, recent *C. gattii* cases have been reported in the southeastern United States (*7*–*9*). We recently described the population structure of 10 *C. gattii* sensu stricto (VGI) patient isolates from the southeastern United States (*6*). Here we describe selecting 8 of those same isolates and using BEAST software (http://beast.community) to predict the timing of the emergence of *C. gattii* in the southeastern United States.

## The Study

We identified single-nucleotide polymorphisms (SNPs) and conducted phylogenetic analyses as previously described (*10*). We identified 43,731 total SNPs among 8 isolates, based on a genome quality breadth of 16,991,136 bases. The maximum-likelihood tree (Figure) had a consistency index of 1.0, and all branching bootstrap values equaled 100 for 100 replicates. Because all isolates in the southeastern United States group contain only the α mating type and the phylogenies have a perfect consistency index (i.e., demonstrating no homoplasy), we assume a clonal expansion for this population (i.e., lack of multiple mating types limits opportunity for recombination), although cryptic recombination cannot be ruled out. We therefore applied the clonal mutation rate obtained from the prior Bayesian molecular clock analysis (using BEAST software [*10*]) of the Pacific Northwest *C. gattii* species complex, specifically for VGIIa and VGIIc (1.59 × 10^−8^ SNPs/base/y). We determined the best-fitting clock and demographic model combination by implementing path and stepping stone sampling marginal-likelihood estimators as described previously (*5*). We implemented a general time-reversible nucleotide substitution model and an uncorrelated log-normal molecular clock with a Bayesian skyline model across 4 chains with 3 billion iterations and achieved across- and within-chain convergence.

**Figure:**
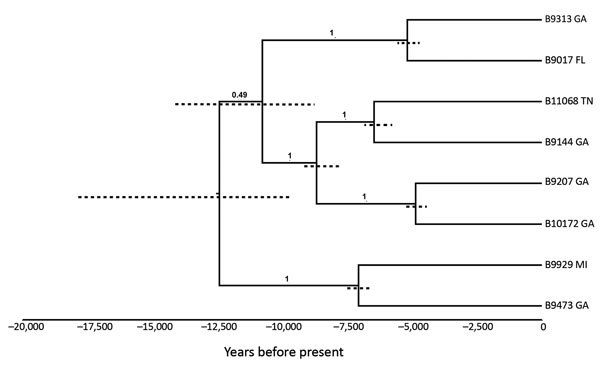
Bayesian phylogenetic analyses of 8 isolates of *Cryptococcus gattii* sensu stricto from the southeastern United States. We used BEAST 1.8.4 software (http://beast.community) to produce calibrated phylogenies with the mean estimates of time to most recent common ancestor. The tips of the branches correspond to the year of sampling. Dotted node bars are shown for each node and indicate 95% CIs for the timing estimate. The timeline represents years before the present day.

Employing these previously described methods with a log-normal Bayesian skyline model on the genomes from the 8 isolates from the southeastern United States, BEAST provided an estimated range of time to most recent common ancestor (tMRCA) of approximately 9,000–19,000 years. A general recombining population mutation rate for *Cryptococcus* was previously estimated at 2.0 × 10^−9^ (*11*), which is an order of magnitude slower than the estimated clonal rate; therefore, applying this rate provides for a tMRCA that is nearly an order of magnitude greater (77,000–148,000 years).

## Conclusions

The clonal mutation rate appears to be more appropriate than the general mutation rate for recombining populations for estimating tMRCA, showing that the timing of *C. gattii* emergence and subsequent divergence in the southeastern United States is clearly much older than the Pacific Northwest emergence. The sharp contrast in the time of arrival compared with *C. deuterogattii* in the Pacific Northwest is considerable. Although *C. gattii* has apparently been in the southeastern United States for thousands more years than *C. deuterogattii* has been in the Pacific Northwest, the number of cases detected in the southeastern United States is far fewer. Infections in the South are probably not regularly missed, given that *C. gattii* in the southeastern United States causes primarily a devastating meningitis or meningoencephalitis (*6*,*9*). Questions of whether subacute cases might be going undetected, whether the distribution of the fungus in the environment might be lower, or whether the niche is not readily accessible by humans remain unanswered. We clearly know less about the *Cryptococcus* species that has been in the United States for thousands of years (*C. gattii* sensu stricto) than we know about the one that has only recently arrived (*C. deuterogattii*).

The Pacific Northwest emergence has been hypothesized to be related to the opening of the Panama Canal, enabling more shipping from eastern ports of South America to the West Coast of North America (*5*). The estimated timing of emergence of *C. gattii* in the southeastern United States occurred long before industrial shipping. Although humans might have populated the southeastern United States by the time of the dispersal, most of the human movement was north to south rather than the reverse (*12*). This relatively recent emergence during the Pleistocene epoch is different from what has been hypothesized for other endemic mycoses, a much older fungal species dispersal resulting from mass migration of animal populations between continents (*13*). The idea of recent emergence also departs sharply from what has been hypothesized as a speciation attributable to prehistoric land movements, as has been proposed for larger *Cryptococcus* species separations (*14*). *C. gattii* sensu stricto has been found in and likely originates from South American locales (*15*). Besides anthropogenic means, possible dispersal mechanisms out of South America might have included animal, bird, or insect species migration or even ocean detritus brought by Caribbean currents or hurricanes. However, given the lack of population structure, dispersal probably occurred through a discrete event or through limited events from the same originating population. No matter the mechanism of arrival, *C. gattii* has been hiding, mostly undetected, in the southeastern United States for millennia.
